# Assessment of bioremediation potential of *Calotropis procera* and *Nerium oleander* for sustainable management of vehicular released metals in roadside soils

**DOI:** 10.1038/s41598-024-58897-9

**Published:** 2024-04-18

**Authors:** Sumreen Anjum, Mubeen Sarwar, Qurban Ali, Muhammad Waqar Alam, Muhammad Tariq Manzoor, Adnan Mukhtar

**Affiliations:** 1 Institute of Botany, Faculty of Life Sciences, University of the Punjab, Lahore, 54590 Pakistan; 2 Department of Horticulture, University of the Punjab, Lahore, 54590 Pakistan; 3 Department of Plant Breeding and Genetics, University of the Punjab, Lahore, 54590 Pakistan; 4 Department of Plant Pathology, University of Okara, Punjab, Pakistan; 5 Department of Plant Pathology, University of the Punjab, Lahore, 54590 Pakistan; 6 Department of Food Science, University of Agriculture, Faisalabad, Suib-Campus Depalpur Okara, Okara, Pakistan

**Keywords:** Heavy metals, *Calotropis procera*, *Nerium oleander*, Traffic density, Sustainable management, Bioremediation, Environmental sciences, Environmental impact

## Abstract

Land transportation is a major source of heavy metal contamination along the roadside, posing significant risks to human health through inhalation, oral ingestion, and dermal contact. Therefore, this study has been designed to determine the concentrations of vehicular released heavy metals (Cd, Pb, Ni, and Cu) in roadside soil and leaves of two commonly growing native plant species (*Calotropis procera* and *Nerium oleander*).Two busy roads i.e., Lahore-Okara road (N-5) and Okara-Faisalabad roads (OFR) in Punjab, Pakistan, were selected for the study. The data were collected from five sites along each road during four seasons. Control samples were collected ~ 50 m away from road. The metal content i.e. lead (Pb), cadmium (Cd) nickel (Ni) and copper (Cu) were determined in the plant leaves and soil by using Atomic Absorption Spectrophotometer (AAS). Significantly high amount of all studied heavy metals were observed in soil and plant leaves along both roads in contrast to control ones. The mean concentration of metals in soil ranged as Cd (2.20–6.83 mg/kg), Pb (4.53–15.29 mg/kg), Ni (29.78–101.26 mg/kg), and Cu (61.68–138.46 mg/kg) and in plant leaves Cd (0.093–0.53 mg/kg), Pb (4.31–16.34 mg/kg), Ni (4.13–16.34 mg/kg) and Cu (2.98–32.74 mg/kg). Among roads, higher metal contamination was noted along N-5 road. Significant temporal variations were also noted in metal contamination along both roads. The order of metal contamination in soil and plant leaves in different seasons was summer > autumn > spring > winter. Furthermore, the metal accumulation potential of *Calotropis procera* was higher than that of *Nerium oleander*. Therefore, for sustainable management of metal contamination, the plantation of *Calotropis procera* is recommended along roadsides.

## Introduction

Heavy metal pollution has become a serious global concern due to heavy metals' toxic, persistent, and non-degradable nature^1^. Pakistan is also afflicted with this problem^2^, which is estimated to become more severe. Vehicular traffic is the environment's key source of heavy metal emissions^3^. Vehicles release these heavy metals via fuel combustion, tire wear and tear, and corrosion of auto-body, engine parts, batteries, radiators, clutches, and brake lining^4^. Cadmium (Cd), copper (Cu), nickel (Ni), and lead (Pb) are the most common vehicular-released metals^5^. These metals contaminate the surrounding environment and adversely affect the roadside flora as they enter the plant body through root or leaf surface^6^, eventually entering animals through food chains. Among these metals, Pb is responsible for human reproductive, renal, and neural disorders^7^. Cadmium is also carcinogenic and may affect reproductive, renal, hepatic, and circulatory systems^8^. Excess of Ni causes dermal, cardiac, and respirational disorders^9^. Exposure to a high concentration of Cu causes gastrointestinal, neurodegenerative, and hepatic disorders in humans^10^.

Heavy metal contamination of the roadside environment is directly linked with traffic density^11^, which is increasing drastically with rapid industrialization and urbanization. The metal distribution along roads also depends on meteorological conditions^12^, road conditions^13^, type of vehicle and fuel used, and driving speed^14,15^. Despite monitoring and controlling all these factors, the extent of heavy metal pollution in roadside surroundings is increasing daily. Therefore, the plant species that can uptake these heavy metals and accumulate them in their different body organs are used, called phytoremediation^16^, which is economical, sustainable, and eco-friendly to protect the ecosystem. Several researchers assessed the metal contamination along roads, but information on traffic-related heavy metal contamination along Lahore-Okara road (N-5) and Okara-Faisalabad road in Punjab, Pakistan is very limited. The metal contamination along these roads may vary spatially and temporally due to heavy traffic loads, which could threaten agriculture. Furthermore, *Calotropis procera* and *Nerium oleander* are among the most commonly grown plant species on these roads. Thus, these plants were selected to monitor the metal contamination along these roads as both are naturally growing along these roads and require very low maintenance.So the present study was planned to: (a) Assess the vehicular released heavy metal contamination in roadside soil. (b) Identify the biomonitor/bioremediator plant species to deal with heavy metal pollution problems. (c) Study the spatial and seasonal variability in heavy metal pollution in roadside environment.

## Material and methods

### Study area

**Figure 1:**
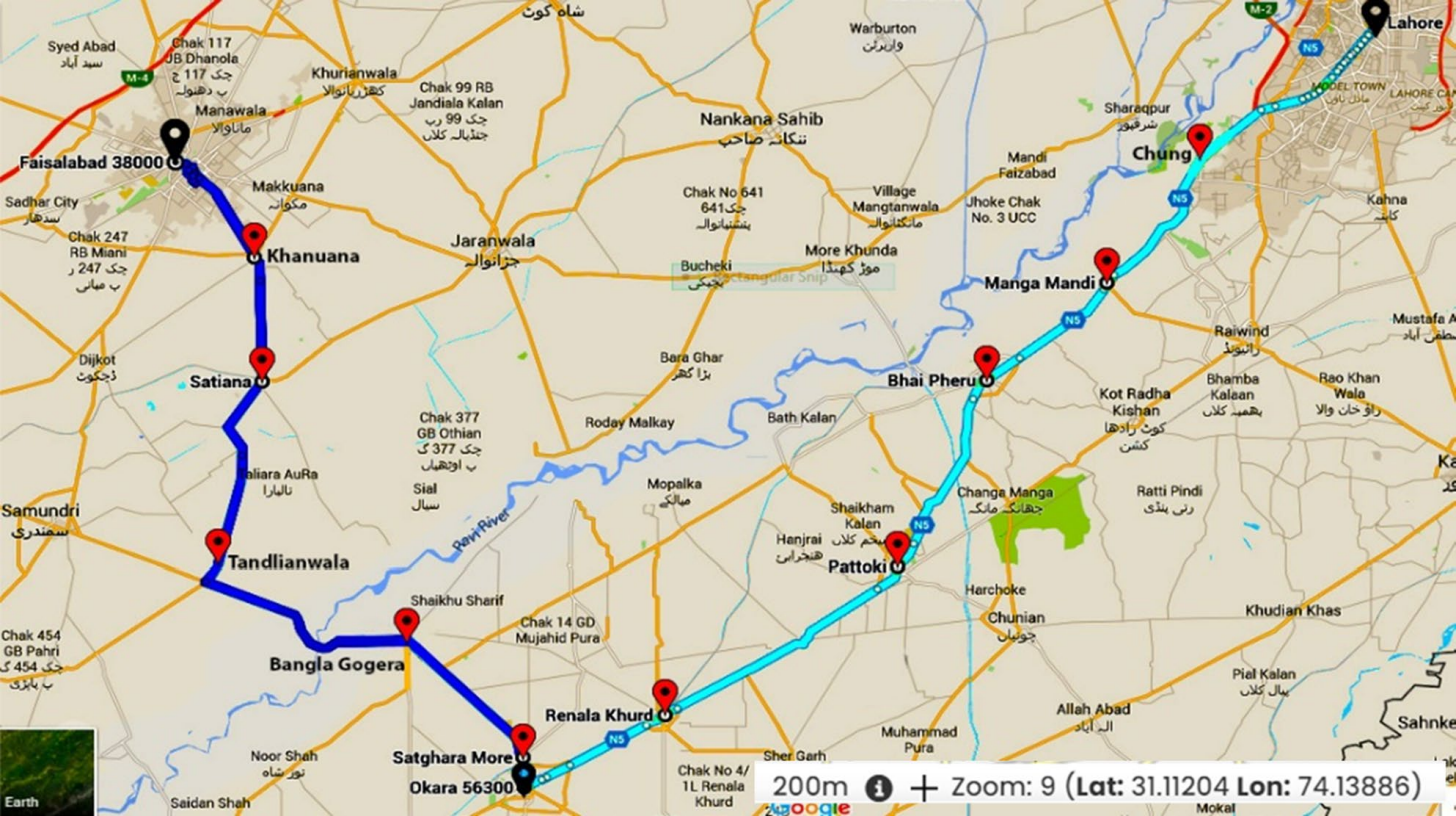
Sampling sites along “N-5” and OFR. Where, (1) Chung; (2) Manga Mandi; (3) Bhai Phero; (4) Pattoki; (5) Renala Khurd; (6) Satghara More; (7) Bangla Gogera; (8) Tandaliawala; (9) Sataiana; (10) Khanuana.

To assess the concentration of traffic-related metals (Cd, Pb, Ni, and Cu,) in roadside soil and bioremediation potential of plants, two busy roads i.e., Lahore-Okara road (N-5) and Okara-Faisalabad roads (OFR) in the Punjab Pakistan, were selected as study area (Fig. 1). Both roads vary in traffic density, vehicle type, and age of roads. The National Highway (N-5) was constructed in 1913 to connect Torkham to Karachi. A very busy section of this highway, Lahore-Okara road (129 km) was selected for study. Five sites (Chung, Manga Mandi, Bhai Phero, Pattoki, and Renala Khurd) were selected. The Okara-Faisalabad road (OFR) is a newly built road with less traffic volume than N-5. Five sites along this road, Satghara More, Bangla Gogera, Tandaliawala, Sataiana, and Khanuana, were selected randomly for the study.

### Sample collection and heavy metal analysis

The case study was conducted in four seasons (summer, autumn, winter, spring). The soil samples and leaves of two commonly growing plant species (*Calotropis procera* A*.,* and *Nerium oleander* L.) were collected from all sites (0–1 m from road edge) of both roads. Control soil and plant leaf samples were collected from 100 m away from road. Five plants of each species from every site were collected during four seasons. Five soil samples were collected from 0–1 m from road edge (0–10 cm deep) from each site along both roads. Vehicular soot (Cars, trucks, and buses) and fuel (diesel, petrol) from various fuel stations along both roads were also analyzed to determine metal contents.

The soil particles on collected leaf samples were removed with deionized distilled water for acid digestion. After drying at 65 °C in a hot-air oven, all samples were milled to powder using Wiley Mill. While, soil samples were first put through a sieve (2-mm) and then dried at 65 °C in an oven for 72 h. Then, using nitric acid and hydrogen peroxide, all air-dried samples (plant leaves and soil) were processed on a hot-block digester by following USEPA, 3050B method for analysis of metals^17^. The methodology of^18^ was followed for soot digestion, and^19^ for petrol and diesel digestion. The Cd, Ni, Pb, and Cu contents in all digested samples were analyzed using an atomic absorption spectrophotometer (AAS). For accuracy and precision in analysis, reference materials with accuracy = 100 ± 20%, reagent blanks, and internal standard solutions were used. Traffic density (average daily traffic/season) was also recorded at all study sites (Table 1). For statistical analyses, analysis of variance was performed with the program COSTAT (Cohort Statistical Software 2003, Monterey, California, USA). Means were compared with LSD test (α = 0.05) to differentiate between different sites, plants, and seasons for metal contamination.

**Table 1:** Average daily traffic (number of vehicles/day) at studied sites.

Roads	N5				OFR				
Sites	Summer	Autumn	Winter	Spring	Sites	Summer	Autumn	Winter	Spring
Chung	24,674.3	23,685.1	19,534.8	22,841.6	Satghara More	13,349.2	13,046.2	9634.3	12,821.3
Manga Mandi	23,721.5	23,148.4	19,076.5	21,836.4	Bangla Gogera	14,863.7	14,327.8	10,648.4	14,714.5
Bhai Phero	23,165.7	22,342.7	18,723.4	22,474.3	Tandaliawala	17,523.2	16,234.3	12,463.8	15,814.1
Pattoki	21,945.6	21,437.8	17,463.6	20,191.8	Sataiana	16,103.3	15,438.5	11,763.4	15,348.1
Renala Khurd	22,467.5	21,856.2	18,214.1	21,734.1	Khanuana	15,484.5	15,137.4	11,846.1	14,853.3

*N-5*  Lahore to Okara road, *OFR*  Okara-Faisalabad road.

### Ethical approval

It has been confirmed that the experimental data collection complied with relevant institutional, national, and international guidelines and legislation with appropriate permissions from authorities of the Department of Horticulture, University of the Punjab, Lahore, Lahore 54300, Pakistan. This research did not contain any studies involving animal or human participants, nor did it occur in any private or protected areas. No specific permissions were required for corresponding locations.

## Results

### Heavy metal content in roadside soil

The mean concentrations of vehicular-released metals (mg/kg) in soils are given in Table 2. Metal (Cd, Pb, Ni, and Cu) content in the soil of all sites along both roads varied significantly. All studied metals were higher in roadside soils than in control site soil. In the present case study, metal content in roadside soil ranged between 2.20 and 6.83 mg/kg Cd, 4.56–15.29 mg/kg Pb, 31.58–101.26 mg/kg Ni and 31.58–101.26 Cu along N-5. Along Okara-Faisalabad road, the Cd, Pb, Ni, and Cu contents were found as 2.73–6.37, 4.53–14.28, 29.78–95.89, and 62.85–132.10 mg/kg, respectively. Among sites, Chung was a highly contaminated site along N-5; along OFR, metal contamination was highest at the Tandliawala site. The comparison of roads showed higher metal contamination along N-5 road. Among seasons, the highest metal contamination in roadside soil was recorded in summer and the least in winter.

**Table 2:** Metal contents (mg/kg) in the roadside soil along N-5 and OFR during different seasons (mean ± SD).

Metals	Roads	Sites	Summer	Autumn	Winter	Spring
Cd	N-5	Control	0.01 ± 0.003 e	0.01 ± 0.003 d	0.004 ± 0.002 g	0.005 ± 0.002 h
		Chung	6.83 ± 0.49 a	6.20 ± 0.23 a	4.16 ± 0.67 ab	5.47 ± 0.40 a
		Manga Mandi	6.05 ± 0.80 b	5.59 ± 0.25 ab	3.71 ± 0.23 bcd	5.11 ± 0.54 abc
		Bhai Phero	6.34 ± 0.18 ab	5.49 ± 0.41 ab	3.34 ± 0.26 cde	4.76 ± 0.18 abcd
		Pattoki	4.24 ± 0.47 d	3.87 ± 0.91 c	2.20 ± 0.39 f	3.34 ± 0.89 g
		Renala Khurd	5.11 ± 0.33 c	4.37 ± 0.47 c	2.98 ± 0.40 de	4.23 ± 0.14 def
	OFR	Satghara More	4.47 ± 0.23 cd	3.91 ± 0.26 c	2.73 ± 0.70 ef	3.42 ± 0.31 fg
		Bangla Gogera	4.56 ± 0.30 cd	4.03 ± 0.38 c	3.38 ± 0.27 cde	3.70 ± 0.65 efg
		Tandaliawala	6.37 ± 0.20 ab	5.62 ± 0.40 ab	4.76 ± 0.21 a	5.12 ± 0.71 ab
		Sataiana	5.82 ± 0.17 b	5.22 ± 0.21 b	4.31 ± 0.42 ab	4.64 ± 0.34 bcd
		Khanuana	5.08 ± 0.40 c	5.37 ± 0.39 b	3.85 ± 0.75 bc	4.30 ± 0.31 cde
Pb	N-5	Control	1.48 ± 0.48 g	0.77 ± 0.15 i	0.45 ± 0.06 f	0.75 ± 0.06 h
		Chung	15.29 ± 0.51 a	13.46 ± 0.94 a	10.75 ± 0.65 a	13.09 ± 0.64 a
		Manga Mandi	13.60 ± 0.58 bc	11.60 ± 0.46 b	9.46 ± 0.66 b	11.78 ± 0.81 b
		Bhai Phero	12.37 ± 0.98 cd	10.21 ± 0.59 bcd	8.19 ± 0.65 c	9.26 ± 0.63 cd
		Pattoki	8.62 ± 0.75 f	5.50 ± 0.38 h	4.56 ± 0.57 e	5.34 ± 0.76
		Renala Khurd	11.75 ± 0.67 de	8.23 ± 0.70 ef	6.51 ± 0.82 d	9.35 ± 0.37 cd
	OFR	Satghara More	9.08 ± 0.43 f	6.27 ± 1.12 gh	4.53 ± 0.45 e	6.46 ± 0.58 g
		Bangla Gogera	10.99 ± 1.43 e	7.03 ± 0.85 fg	5.50 ± 0.52 de	7.32 ± 1.18 ef
		Tandaliawala	14.28 ± 0.39 ab	11.30 ± 1.18 bc	8.15 ± 0.59 c	10.34 ± 1.02 c
		Sataiana	12.48 ± 0.85 cd	9.87 ± 1.39 cd	6.37 ± 1.39 d	8.18 ± 1.05 de
		Khanuana	11.44 ± 0.83 de	8.97 ± 0.95 de	5.94 ± 0.17 d	7.61 ± 0.64 ef
Ni	N-5	Control	34.94 ± 5.50 f	31.61 ± 12.76 f	21.35 ± 4.02 e	32.95 ± 3.40 d
		Chung	101.26 ± 12.49 a	93.20 ± 6.70 a	72.80 ± 13.35 a	87.90 ± 14.12 a
		Manga Mandi	94.49 ± 8.81 ab	76.69 ± 6.80 bc	59.56 ± 18.03 ab	78.71 ± 10.69 a
		Bhai Phero	82.25 ± 6.55 c	88.85 ± 4.25 a	39.59 ± 9.53 cd	80.21 ± 2.87 a
		Pattoki	51.38 ± 5.27 e	40.46 ± 10.07 ef	31.58 ± 6.19 de	39.88 ± 6.26 cd
		Renala Khurd	80.67 ± 7.73 cd	60.62 ± 11.92 d	36.39 ± 12.04 cde	43.63 ± 7.00 cd
	OFR	Satghara More	54.45 ± 12.49 e	46.11 ± 6.70 e	34.04 ± 13.35 cde	36.53 ± 14.12 cd
		Bangla Gogera	69.92 ± 8.81 d	65.27 ± 6.80 cd	29.78 ± 18.03 de	45.27 ± 10.69 c
		Tandaliawala	95.89 ± 6.55 ab	84.83 ± 4.25 ab	60.06 ± 9.53 ab	77.87 ± 2.87 a
		Sataiana	84.94 ± 5.27 bc	75.52 ± 10.07 bc	51.42 ± 6.19 bc	62.19 ± 6.26 b
		Khanuana	77.83 ± 7.73 cd	62.31 ± 11.92 d	45.45 ± 12.04 bcd	64.81 ± 7.0 b
Cu	N-5	Control	57.33 ± 4.42 h	61.97 ± 4.81 f	48.05 ± 7.22 e	60.73 ± 4.78 e
		Chung	138.46 ± 10.26 a	129.71 ± 6.17 a	111.38 ± 7.54 a	113.39 ± 9.52 a
		Manga Mandi	128.57 ± 11.71 abc	123.22 ± 9.87 a	102.86 ± 11.31 a	104.14 ± 6.79 ab
		Bhai Phero	119.63 ± 6.13 bcd	107.85 ± 4.95 bc	88.91 ± 4.47 b	104.11 ± 12.34 ab
		Pattoki	82.39 ± 7.10 g	73.08 ± 6.93 ef	61.68 ± 8.54 d	75.87 ± 3.51 d
		Renala Khurd	115.95 ± 9.07 cde	96.43 ± 7.92 d	73.94 ± 10.35 cd	94.09 ± 10.44 bc
	OFR	Satghara More	94.54 ± 10.26 fg	80.91 ± 6.17 e	65.15 ± 7.54 cd	79.21 ± 9.52 cd
		Bangla Gogera	104.73 ± 11.71 ef	94.39 ± 9.87 d	62.85 ± 11.31 d	87.76 ± 6.79 cd
		Tandaliawala	132.10 ± 6.13 h	124.16 ± 4.95 a	104.38 ± 4.47 a	111.23 ± 12.34 a
		Sataiana	122.53 ± 7.10 bc	109.47 ± 6.93 b	89.66 ± 8.54 b	94.82 ± 3.51 bc
		Khanuana	109.23 ± 9.07 de	97.25 ± 7.92 cd	78.19 ± 10.35 bc	94.24 ± 10.44 bc

*N-5*  Lahore to Okara road, *OFR*  Okara-Faisalabad road.

Letters ‘abcdefg’ represent significant difference among sites. Same letters on different values signify non-significant differences (Fisher's LSD 0.05).

### Bioremediation potential of roadside plants

**Figure 2:**
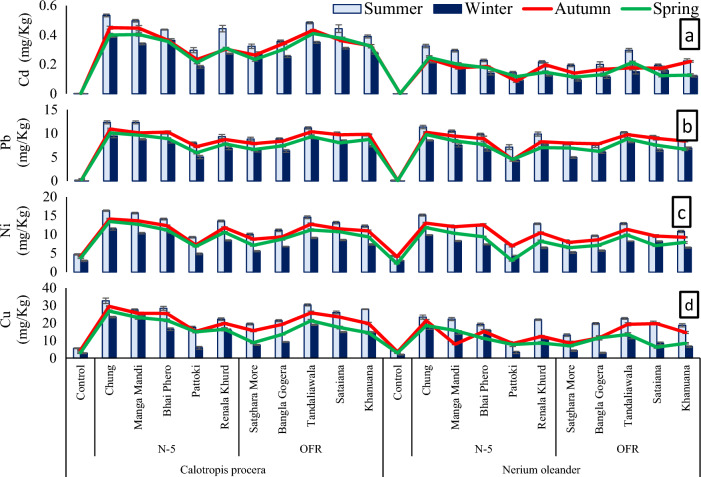
Metal content in plants leaves collected from various sites along N-5 and OFR during different seasons where, (**a**) Cd contents in plant leaves, (**b**) Pb contents in plant leaves, (**c**) Ni contents in plant leaves, (**d**) Cu contents in plant leaves. *N-5* Lahore to Okara road, *OFR* Okara-Faisalabad road.

**Figure 3:**
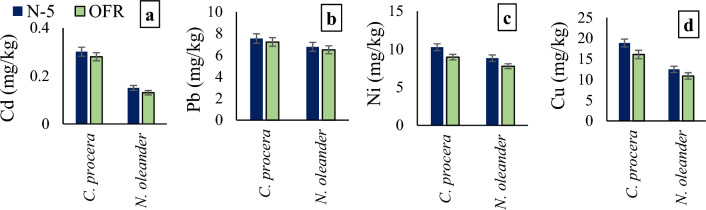
Metal uptake in plant leaves where, (**a**) Cd contents in plant leaves, (**b**) Pb contents in plant leaves, (**c**) Ni contents in plant leaves, (**d**) Cu contents in plant leaves. *N-5*  Lahore to Okara road, *OFR * Okara-Faisalabad road.

**Figure 4:**
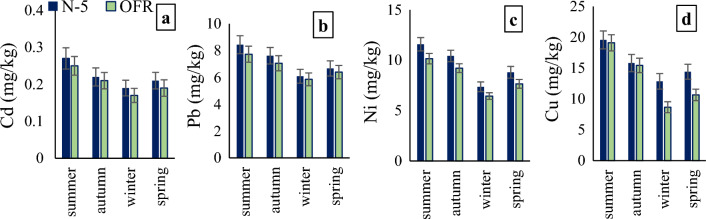
Metal uptake in plants leaves collected from various sites along N-5 and OFR during different seasons where, (**a**) Cd contents in plant leaves, (**b**) Pb contents in plant leaves, (**c**) Ni contents in plant leaves, (**d**) Cu contents in plant leaves. *N-5*  Lahore to Okara road, *OFR*  Okara-Faisalabad road.

The amount of metals (Cd, Pb, Ni, and Cu) in roadside plant leaves was considerably higher than the control site plants during all seasons. Among sites, the maximum amount of metal in plant leaves was observed at Chung site along N-5 and Tandaliawala site along OFR (Fig. 2). The mean metal uptake in *Calotropis procera* was higher than *Nerium oleander* (Fig. 3). Among seasons, plant metal content was highest in the summer season (Fig. 4).

### Metal content in fuel and soot

High amounts of Cd, Pb, Ni, and Cu were noted in diesel, petrol, and used motor oil (Table 3). The categorization of metal content in fuel was Cd < Pb < Ni < Cu. However, the metal contents in used motor oil were highest. High metal content was also found in the soot of various vehicles, though truck soot has the highest amount of Cd, Pb, Ni, and Cu i.e., 0.97, 8.55, 12.78, and 21.51 mg/kg, respectively.

**Table 3:** Metal content in fuel (petrol and diesel), used motor oil and soot (mean ± S.D.).

	Petrol (mg/L)	Diesel (mg/L)	Used motor oil (mg/L)	Soot of trucks (mg/kg)	Soot of bus (mg/kg)	Soot of car (mg/kg)
Cd	0.083 ± 0.006	0.074 ± 0.003	0.624 ± 0.032	0.971 ± 0.046	0.746 ± 0.068	0.158 ± 0.030
Pb	0.382 ± 0.01	0.304 ± 0.012	6.317 ± 0.380	8.853 ± 0.453	6.369 ± 0.321	2.473 ± 0.219
Ni	4.477 ± 0.489	2.501 ± 0.158	5.872 ± 0.325	12.78 ± 0.211	12.32 ± 1.335	6.891 ± 0.161
Cu	6.740 ± 0.272	6.223 ± 0.109	16.18 ± 0.438	21.51 ± 0.795	17.66 ± 0.681	9.487 ± 0.472

## Discussion

### Heavy metal content in roadside soil

The mean concentrations of vehicular-released metals (mg/kg) in soils have been presented in Table 2. Significant differences existed in metal (Cd, Pb, Ni and Cu) content in the soil of all sites along both roads. All studied metals were higher in roadside soils than in control site soil. Among sites, all the metals were higher in concentration at Chung site along N-5, and along OFR it was highest at Tandliawala site. Among seasons, the highest metal contamination in roadside soil was recorded in summer and the least in winter. The roads with high traffic volume and speedy automobiles incorporate many metals into the environment that sooner or later get deposited on the roadside soil^20^. During this study, high metal contents were found along N-5 as compared to ORF. Many earlier studies also found higher metal contamination in roadside soils^15,21–23^.

Cadmium (Cd) in roadside environment come from burning fuel, corrosion of radiators and batteries, and wearing old tires^24^. In the present case study, Cd content in roadside soil ranged between 2.20 and 6.83 mg kg^−1^ along N-5 and 2.73–6.37 mg kg^−1^ along Okara-Faisalabad road. Many earlier researchers also found higher Cd content in roadside soil^15,25, 26^.

Lead (Pb) is a most important autoexhaust pollutant ^27^. Regardless of the ban on leaded fuel, it is still found in petrol. In the current study, the content of Pb in roadside soil was significantly higher in contrast to control site soil. These results conform with many earlier studies ^26,28–31^. The mean Pb content in roadside soil along N-5 ranged between 4.56 and 15.29 mg/kg, and along Okara-Faisalabad road it ranged 4.53–14.28 mg/kg. These results showed that the vehicles are the key source of Pb contamination in roadside soil^32^.

Nickel (Ni) concentration in roadside soil was significantly higher than control site soil. The mean Ni content in roadside soil along N-5 ranged between 31.58 and 101.26 mg/kg, and along Okara-Faisalabad road it ranged 29.78–95.89 mg/kg. Many earlier researchers also reported higher levels of metals in roadside soil^33,34^. The Ni in roadside soil might have come from engine oil, fuel, tire, and brake wear ^12^ and corrosion of nickel alloy bearings, valves, and shafts ^35^.

The amount of Cu in roadside soil (61.68–138.46 mg/kg along N-5 and 62.85–132.10 mg/kg along Okara-Faisalabad road) was significantly higher than in control site soil. These results parallel many former studies^36–38^. This showed that the traffic is one of the major sources of Cu contamination in soil ^39^. Automobiles released Cu into the surrounding environment from fuel burning, battery corrosion^38^ and brakes and tire wear^40^. The metal content (Cd, Pb, Ni and Cu) found during present study was higher than the permissible limit of these metals in the soil set by ^41^(Table 4).

**Table 4:** Permissible limits of heavy metal contents (mg/kg) in soil and plants.

	Cd (mg/kg)	Pb (mg/kg)	Ni (mg/kg)	Cu (mg/kg)	References
Soil	0.4–1.0	3.0 (WHO, 1996)	15–50	40–75	ECDGE, 2010^41^
Plants	0.02	2.0	10	10	WHO,^42^; Lone et al.,^43^

### Heavy metal content in roadside plants

Plants that grow on heavy metal-polluted soil uptake those metals along with other essential minerals and accumulate them in different body parts^16^. The metal content (Cd, Pb, Ni, and Cu) found along N-5 and OFR were above the permissible limit of these metals in the plants set by WHO (Table 4). Compared to common plant species, metal accumulating plants typically exhibit unique and distinct compartmentation and build different complexes^44^. Metals like cadmium induces phytochelatins (PCs) formation in plants; however, it is bound by weak oxygen ligands as a substitute for strong sulfur ligands in metal hyperaccumulators^45^. This is distinctive of many metal accumulating plants^44^. In plants, the accumulation of different metals is also influenced by environmental factors and plant genotypes^46^. So, it is different in different plants, even under the same environments^47^. Plant physiological mechanisms play an important role in metal accumulating plants to alleviate metal toxicity. Cell binds the metal with the cell wall and immobilizes it, thus alleviating metal toxicity in plants^48^. Furthermore, metal that enters the cell bonds to various organic ligands to produce stable chelates that are subsequently taken up by the vacuole^49^. In the current study, the metal accumulation potential of *Calotropis procera* was significantly higher than that *Nerium oleander*. So, both plants could be used to monitor metal contamination, while Calotropis procera could be a better choice to remediate the metal-contaminated soil.

### Spatial variations in heavy metal contents in roadside soil and plant leaves

The difference in metal contamination at different sites is directly related to the traffic density of that particular site. Higher metal content in soil and plant leaves at Chung and Tandaliawala was due to high traffic flow at these sites^11^. Furthermore, Pattoki site along N-5 was the least contaminated site among all other sites. This is because of high vegetation protection along this site, as many plants can naturally ameliorate metal-contaminated areas^50^. The higher concentrations of metals along N-5 are attributed to higher traffic volume along this road. Besides traffic density, several other factors i.e., road structure, type of vehicles, fuel, and age of roads^51^, also contribute to metal contamination near roads. The national highway (N-5) is a very old concrete road, and older roads retain significant amount of deposited metals in nearby soil^26^. Furthermore, concrete roads cause more metal contamination than asphalt roads^52^. The speed of vehicles along N-5 remains very high compared to OFR, which causes more tire wear and tear, resulting in high metal content in the surrounding environment^26^. The reason for less metal content along OFR is that it is newly constructed and less busy than N-5 road. However, high metal contents were recorded at the Tandaliawala site, which act as junctions/temporary bus stops for passenger vans. This is supported by the results of^53^, who found high metal content at traffic junctions and crossroads.

### Seasonal variation in heavy metal content in roadside soil and plant leaves

Metal contamination in roadside soil and plant leaves was higher in the summer and lowest in winter. The high metal contamination in summer might be due to high traffic density and more rubber abrasion at high temperatures (average day temperature is 43.5 °C). Furthermore, metals are more bioavailable at high temperatures, and plant accumulation is higher in plants during summer^3^. Metals percolate deep into the soil due to heavy precipitation during late summer ^54,56, 57^ also observed seasonal variation in metal contamination.

### Metal content in fuel and soot

Despite the ban on leaded fuel, Pb was still found in petrol, diesel, and used motors during the current investigation. Lead-containing petrol is always considered a major cause of Pb pollution. Metal content in automobiles’ fuel found during the present study was greater than those reported by^55^. They noted 0.04 μg/g Cd, 4.50 μg/g Pb, 0.22 μg/g Ni, and 7.00 μg/g Cu in used motor oil, while Cd and Cu were not detectable in unused oil and Pb was lower in concentration (2.00 μg/g) in unused oil. This indicates that metals are released from automobiles during different process ^3,35^ also reported metal content in soot of vehicles. The higher amount of metals in fuel and soot of vehicles proved our findings that the vehicles are the main contributors of metal pollutants in the environment.

## Conclusion

Heavy metal (Cd, Pb, Ni, and Cu) concentrations in roadside soil and plant leaves were much higher than the standard permissible limits. They showed a strong positive connection with traffic density. This demonstrates that automobiles are the primary source of heavy metal contamination in the environment near roadsides. Residents living close to roads and agriculture may suffer as a result. For control to be effective, heavy metal contamination must be regularly monitored. The current study focuses on employing native plant species to reduce soil contamination with Cd, Pb, Ni, and Cu as a sustainable management strategy. *Calotropis procera* is a useful metal accumulator. Therefore, it could be used to reclaim the heavy metal-contaminated soils. Though this study offers valuable insights, it is imperative to recognize its limits. The study locations chosen are urban areas, and more research in other locations and climates could enhance the conclusions' generalizability. Furthermore, long-term monitoring and assessing their performance would provide a more thorough knowledge of the chosen tree species' potential as useful instruments for improving environmental quality. Altogether, our research highlights the critical role that native vegetation plays in reducing environmental pollution and offers insightful advice to environmentalists, legislators, and urban planners on how to create more sustainable and healthy urban settings. We can get one step closer to creating more resilient and environmentally friendly cities for the benefit of present and future generations by incorporating suitable tree species into urban landscapes.

## Data Availability

The datasets used and/or analyzed during the current study has been available in the manuscript.
